# Control of Meniscus Formation Using an Electrohydrodynamics Module in Roll-to-Roll Systems for the Stable Coating of Functional Layers

**DOI:** 10.3390/polym16060845

**Published:** 2024-03-19

**Authors:** Minjae Kim, Minho Jo, Jaehyun Noh, Sangbin Lee, Junyoung Yun, Gyoujin Cho, Changwoo Lee

**Affiliations:** 1 Department of Mechanical Design and Production Engineering, Konkuk University, 120 Neungdong-ro, Gwangjin-gu, Seoul 05029, Republic of Korea; kimalswo@konkuk.ac.kr (M.K.); als8080@konkuk.ac.kr (M.J.); zenty616@konkuk.ac.kr (J.N.); tkdqls2052@konkuk.ac.kr (S.L.); jun980520@konkuk.ac.kr (J.Y.); 2 Institute of Quantum Biophysics, Sungkyunkwan University, 2066 Seobu-ro, Jangan-gu, Suwon 16419, Republic of Korea; gcho1004@skku.edu; 3 Department of Mechanical Engineering, Konkuk University, 120 Neungdong-ro, Gwangjin-gu, Seoul 05029, Republic of Korea

**Keywords:** coating meniscus, electrohydrodynamics, functional layer, roll-to-roll processing, slot-die coating, spreadability control, stable coating, thickness uniformity

## Abstract

In fabricating functional layers, including thin-film transistors and conductive electrodes, using roll-to-roll (R2R) processing on polymer-based PET film, the instability of the slot-die coating meniscus under a high-speed web impedes functional layer formation with the desired thickness and width. The thickness profiles of the functional layers significantly impact the performance of the final products. In this study, we introduce an electrohydrodynamic (EHD)-based voltage application module to a slot-die coater to ensure the uniformity of the cross-machine direction (CMD) thickness profile within the functional layer and enable a stable, high-speed R2R process. The module can effectively control the spreadability of the meniscus by utilizing variations in the surface tension of the ink. The effectiveness of the EHD module was experimentally verified by applying a high voltage to a slot-die coater while keeping other process variables constant. As the applied voltage increases, the CMD thickness deviation reduces by 64.5%, and the production rate significantly increases (up to 300%), owing to the formation of a stable coated layer. The introduction of the EHD-based application module to the slot-die coater effectively controlled the spreadability of the meniscus, producing large-area functional layers.

## 1. Introduction

Roll-to-roll (R2R) processing is an efficient continuous production method that enables large-area, high-speed manufacturing. It is particularly suited for coating a uniform functional layer onto a thin web. Moreover, R2R processing is environmentally friendly, simple, and low-cost. Thus, R2R processing has recently garnered considerable attention across diverse fields, such as printed electronics, including printed photovoltaics; organic thin film transistors (TFTs); capacitors; and coating the water-repellent solution of solar cells. It is also of great interest in the battery industry, such as in the manufacture of lithium-ion battery electrodes, solid oxide fuel cells, and battery separators based on polyvinylidene fluoride [1,2,3,4,5,6,7,8]. In the fabrication of most common electronic devices using R2R continuous systems, the thickness of the coated or printed functional layer has a significant effect on the electrical performance of the product [9]. For instance, the structure of a fully R2R printed capacitor has an upper conductive layer, an intermediate dielectric layer, and a lower conductive layer; additionally, the machine direction (MD) and cross-machine direction (CMD) thicknesses of the dielectric layer formed by slot-die coating determine the capacitance of the capacitor [10]. Furthermore, in the fabrication of electrodes, the MD and CMD thickness profiles of the electrodes affect the short-circuit potential of the battery and are crucial in determining the number of composite layers of the anode, separator, and cathode that can be packed into a cell pouch [11,12,13,14]. The thickness profile deviation can lead to defects that affect the performance of the functional layer or reduce the ease of subsequent processes. To prevent this, it is crucial to conduct process optimization in advance.

Furthermore, in response to the rapidly growing EV battery market, electrodes and separator layers are being increasingly manufactured via R2R processing to achieve high production volumes [15,16,17]. The operating speed of the R2R process is highly dependent on the speed of the web transportation (web speed). However, the web speed cannot be increased indefinitely for several reasons, which decreases production output [18]. For example, if the web speed is increased excessively, the geometry of the functional layers and patterns being coated or printed would differ from the desired geometry [19]. When the web passes over the lower part of the meniscus formed at the slot-die coater lip at a high speed, a large shear force is generated between the ink and the web, and the ink is coated discretely in the form of a strip, instead of being coated continuously with the CMD as desired [20]. Thus, the meniscus of the slot-die coater becomes unstable under high-speed conditions, making it difficult to form a functional layer with the desired thickness and width.

Numerous studies have been conducted to address the above problems, ensuring coated layer thickness uniformity and coating stability. Kang et al. analyzed the sensitivity of process variables, including the velocity, gap, and flow rate, to the thickness of the coating layer and selected optimal operating conditions based on the results of the analysis [21]. Park et al. analyzed the impact of coating speed and changes in the coating bead on the deviation of the width-direction thickness and successfully minimized the deviation by controlling these factors [22]. Additionally, Huang et al. conducted performance optimization by considering the variation in the thickness of the functional layer when adjusting the amount of solvent additive and the processing temperature [23]. However, most studies have solely focused on minimizing the thickness deviation in the MD. Even those studies that address thickness deviation in the CMD may not be conducive to ensuring coating stability and uniformity through real-time control during the coating process, as they often necessitate alterations to the process conditions of the slot-die coater or adjustments to the solution itself. Furthermore, there exists a controllable upper limit through previously studied methods, and it is crucial to explore new module applications to achieve higher productivity beyond the established threshold.

Therefore, this study aimed to uniformize the CMD thickness profile of functional layers produced by slot-die coating and ensure stable high-speed R2R processing. A slot-die coater was combined with an electrohydrodynamic (EHD)-based voltage application module to control the spreadability of the CMD and MD meniscus. To achieve a level of stability that cannot be attained by adjusting the process parameters of the slot-die system, this control method utilizes additional energy based on the formation of an electric field to assist in leveling the ink [24]. Extant research on EHD inkjet printing has primarily focused on controlling the surface tension of the meniscus in a physical–electrical manner, formed by applying a high voltage to the ink. This approach has been centered on achieving fine linewidth printing at the nanoscale [25,26,27]. However, these studies have faced challenges in production speed, preventing them from being applied to address the mass production demand of high-growth industries. In this study, the EHD principle is applied to a slot-die coater to control the spreadability of the meniscus in producing large-area functional layers, which considerably improved the CMD thickness uniformity and coating stability at high speeds. Furthermore, the effectiveness of the proposed method is experimentally demonstrated to an extent for both conductive and non-conductive inks.

## 2. Theoretical Background

### 2.1. Roll-to-Roll Slot-Die Coating Systems

**Figure 1:**
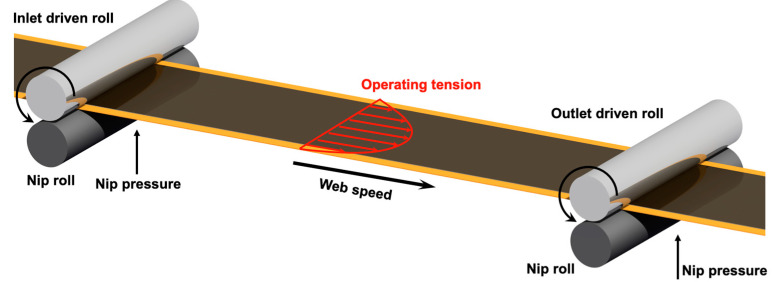
Span configuration in a roll-to-roll (R2R) system.

**Figure 2:**
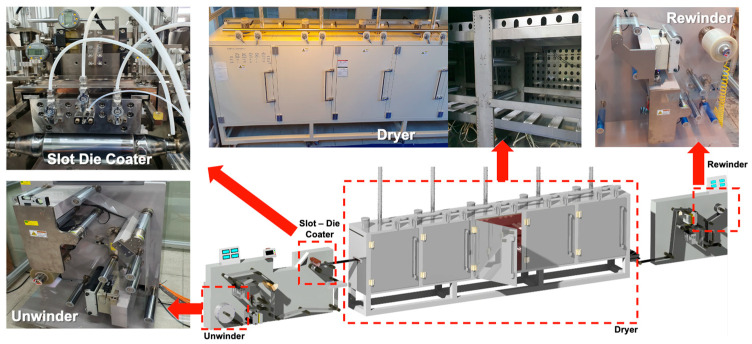
Schematics of R2R continuous slot-die coating system.

The R2R system is typically composed of driven rolls, which are driven by a motor, and idle rolls, which are non-driven rolls. The space between two driven rolls is defined as a span [28]. A span has one driven roll at the inlet and one at the outlet. As shown in Figure 1, the driven roll is typically paired with a nip roll, which presses the web with a strong nipping pressure while rotating to transfer the web [29]. The R2R system is obtained from a configuration of several spans, as illustrated in Figure 2.

The R2R system used in this study (Figure 2) comprised four broad sections: unwinding, coating, drying, and rewinding sections. In the unwinding section, the web is gradually unwound in a wound roll. In the coating section, a functional layer is coated on the continuously conveyed web through a slot-die coater. The functional layer coated in the liquid state enters the drying section to evaporate the solvent, and the functional layer in the solid state after the solvent evaporates is wound again in the form of a wound roll in the rewinding section. The process parameters in all sections, except the unwinding section, influence the thickness of the coated functional layer. In the coating section, several factors—such as the ink discharge rate, the meniscus formed between the coater lip and the web, and the contact angle between the ink and the web—have a significant impact on the thickness of the coated functional layer. A detailed description is provided in a later section. In the drying section, the thickness of the functional layer is affected by the coffee ring effect, in which the solvent is concentrated on the edge of the droplet as the solvent evaporates, as well as the CMD and MD temperature deviations of the web inside the dryer [30,31]. In the rewinding section, the web is wound in a tension profile that gradually decreases with time. The tension profile is defined as a taper tension profile, and the wound roll has an internal stress distribution in the circumferential and radial directions [32,33,34]. Consequently, the coated functional layer is subjected to a strong nipping pressure, owing to the shape of the wound roll, reducing the thickness.

Furthermore, from the overall system perspective, the tension on the web owing to the difference in the speeds of the drive rolls causes a CMD distribution, and the CMD of the web reduces the tension at the ends compared to the tension at the center (Figure 2) [35]. At this point, the surface roughness of the web is reduced in the area where high tension is applied compared to that in the area where low tension is applied, which reduces the contact angle between the ink and the web [36]. This tension-induced variation in the contact angle between the ink and the web changes the CMD thickness profile of the functional layer.

Although several factors in the R2R slot-die-coating system vary the thickness of the coated functional layer, we focused on the functional layer CMD thickness variation that occurs in the coating section [37,38,39]. To mitigate this variation, we first analyzed the reasons for the thickness variation of the coated functional layer in the coating section.

### 2.2. Coated Layer Thickness Uniformity

**Figure 3:**
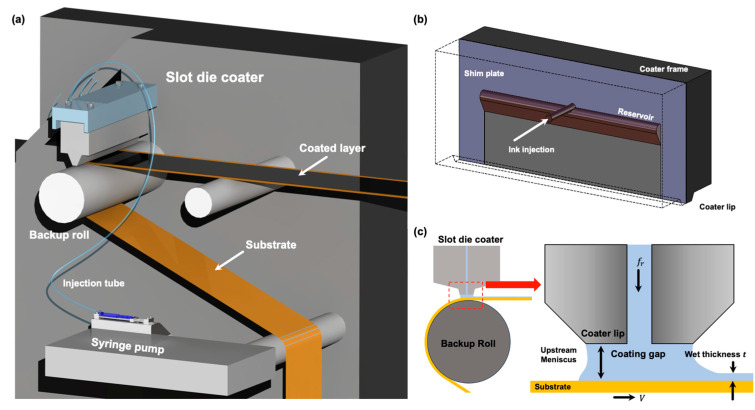
Schematics of (**a**) R2R continuous coating section, (**b**) slot-die coater, and (**c**) coater lip detailed view.

Figure 3a shows a simplified schematic of a slot-die coating. A backup roll is placed at the bottom of the slot-die coater to transfer the web, and the functional layer is coated on the backup roll. The ink for the functional layer is injected into the slot-die coater using a syringe line mounted on a syringe pump. The ink is used to fill the reservoir of the slot-die coater, as illustrated in Figure 3b. Once the reservoir is filled, it is ejected from the coater lip through the gap of the shim plate and used to coat the conveyed web. The thickness of the coating layer formed by the ink ejected from the coater lip can be predicted using a continuous equation [40,41]. When the law of conservation of mass is applied to the coater lip illustrated in Figure 3c, the following equation is obtained:

(1) $$\frac{d}{dt}\int V\left(x\right)dx=f_{r}-[wtV]$$

where $V\left(x\right)$ indicates the mass of the control volume, $f_{r}$ denotes the flow rate of the fed ink, w denotes the width of the coated layer, t indicates the thickness of the coated layer, and V indicates the speed of the web transferred through the backup roll from the bottom of the slot-die coater. In the normal state, where no change occurs over time, Equation (1) can be expressed as follows:

(2) $$f_{r}=wtV$$

It can be observed from Equation (2) that the thickness of the coated layer is influenced by the feed flow rate of the ink discharged from the coater lip, the width of the coated layer, and the speed of the web. Here, the speed of the ink ejected from the coater lip forms the outlet velocity profile with the CMD owing to the wall condition of the fluid flowing on the coater wall. When Equation (2) is expanded to three dimensions by considering the dimension of width, it can be inferred that the coating layer forms a different wet thickness with the CMD because the ink ejection velocity differs at individual points of the CMD. To verify this inference, we modeled the dynamics of the ink inside the slot-die coater using computational fluid dynamics (ANSYS V18.0, Canonsburg, PA, USA). Accordingly, the outlet velocity of the coater lip was obtained. The governing equations of the ink flow inside the slot-die coater are expressed as Equations (3) and (4) and are based on the Navier–Stokes equations, which contain continuous equations and incompressible equations of a viscous fluid [42]. Here, ρ, t, V, g, and μ represent density, time, fluid velocity, gravitational acceleration, and viscosity coefficient, respectively.

(3) $$\frac{\partial \rho }{\partial t}+\nabla \cdot \left(\rho V\right)=0$$

(4) $$\rho \left[\frac{\partial V}{\partial t}+\left(V\cdot \nabla \right)V\right]=-\nabla p+\rho g+\mu \nabla^{2}V$$

The flow resistance of a viscous fluid can be expressed as Equation (5), where σ, η, $\dot{e}$, and $\dot{\gamma }$ represent the stress applied to the element, viscosity of the fluid, strain rate of the element with σ, and nominal shear strain, respectively.

(5) $$\sigma =2\eta \dot{e}=\eta \dot{\gamma }$$

**Figure 4:**
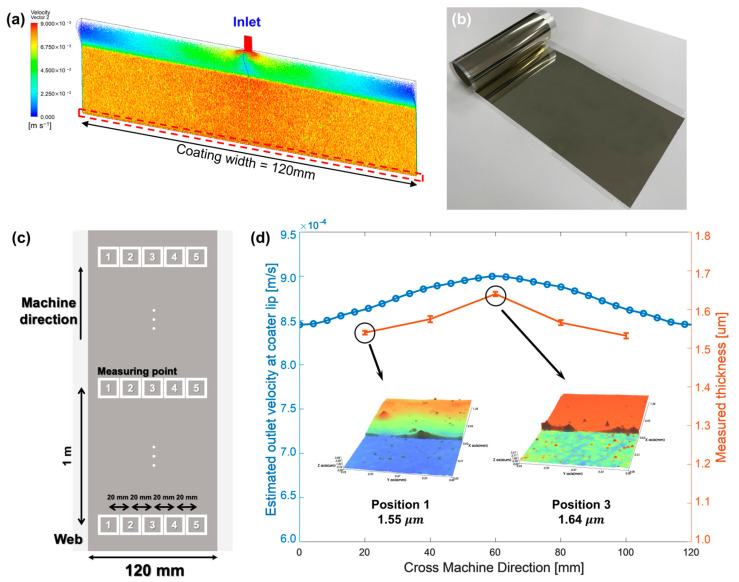
CMD thickness variation due to outlet velocity deviation of coater lip: (**a**) CFD simulation for estimating the outlet velocity profile, (**b**) PET substrate-based silver coating layer, (**c**) Measurement points for coated layer thickness, and (**d**) correlation between estimated outlet velocity profile and measured thickness of the silver-coated layer.

Upon fabricating the silver-ink-coated layer using slot-die coating, the correlation between the outlet velocity of the coater lip predicted using CFD and the measured thickness of the silver-ink-coated layer was verified. Figure 4a,b illustrates the flow of ink inside the coater through the velocity vector and the silver-ink-coated layer fabricated by the slot-die coater, respectively. Table 1 summarizes the physical properties and boundary conditions of the ink used in the simulations and experiments. The thickness of the coated layer was measured as shown in Figure 4c. The thickness measurements of the coated layer were conducted at two points on each side at 20 mm intervals from the center, for a total of five points, and at three points at 1 m intervals in the machine direction. The coated layer thickness measured in the width direction and the fluid velocity profile at the coater lip are graphically represented in Figure 4d. The ink ejected from the center of the coater lip has a higher velocity compared to the ink ejected from both ends, owing to the friction of the walls inside the coater. Although a colloidal-based solution was used as the ink, it was rapidly dried using a drying system with an infrared radiation lamp, which allows the coffee ring effect to be neglected [43]. Consequently, the CMD velocity variation of the ink at the coater lip exhibits a similar trend to the CMD thickness variation of the coated layer.

The CMD thickness profile of the coated layer formed through slot-die coating becomes non-uniform due to the deviation in the outlet velocity at the coater lip. When we apply this result by considering the ink supply flow rate in Equation (2), the CMD wet thickness variation of the coated layer is induced. This thickness variation of the coated layer causes serious defects in many applications. For instance, during the multilayer printing of TFTs, the thickness of the active layer determines the threshold voltage variation of the TFT [44]. If the active layer thickness of each TFT varies with the CMD, the performance of TFTs printed on a single web may be changed by the CMD. Moreover, the electrodes of EV batteries manufactured via the R2R process involve coating an active material onto a copper or aluminum foil using slot-die coating. During this process, the non-uniformity in the thickness of the active material significantly impacts interfacial stability and long-term cycling performance [45]. Additionally, when produced in a winding form through a winding process, the concentration of winding stresses in relatively thick sections can damage the electrode layer. Therefore, the CMD thickness variation of the coated functional layer is a problem that must be mitigated. One way to mitigate this problem in the form of increased wet thickness at the center compared to the two ends involves increasing the spreadability of coated ink on the center of the web and moving the ink to the two ends.

### 2.3. Instability at High Operating Speeds

Another major problem with R2R systems is the difficulty in maintaining stable coating performance during high-speed production. Ruschak proposed Equation (6) to predict the minimum thickness required to maintain a stable coating bead [46]. In this equation, the Capillary number (Ca) is defined as the ratio of the liquid’s viscosity to the surface tension, as shown in Equation (7).

(6) $${th}_{w}=\frac{H_{0}}{\left(\frac{2}{1.34{Ca}^{2/3}}+1\right)}$$

(7) $$Ca=\frac{\mu V}{\sigma }$$

Here, μ represents the viscosity of the ink and V denotes the web speed. Equation (6) reveals that the thickness varies due to the maximum pressure gradient between the meniscus and air. According to this model, as the transfer speed of the material increases, and the stability of the coating decreases, necessitating a greater wet coating thickness to achieve the minimum flow rate for meniscus stability.

**Figure 5:**
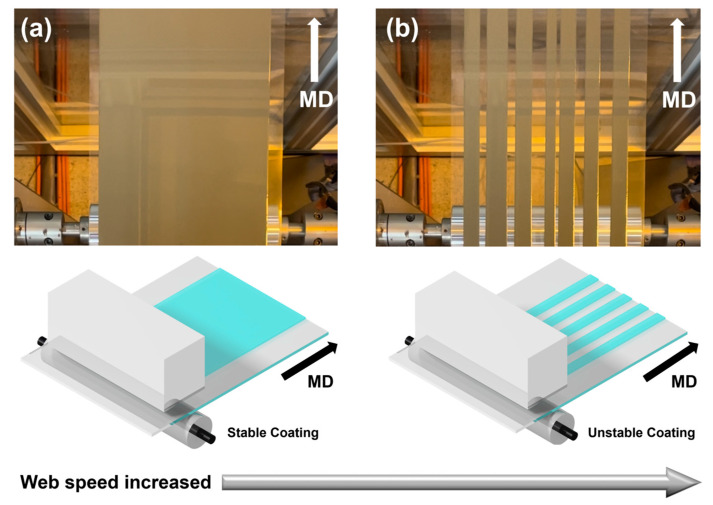
Formation of (**a**) coated layer at proper web speed and (**b**) strip-coated layer owing to excessive web speed.

If the production speed is excessively increased to increase the output, the flow rate of ink discharged to the coater lip will struggle to match the speed of the conveyed web from the bottom of the coater, breaking the coating beads and resulting in a strip-like coating layer with an irregular uncoated CMD, as shown in Figure 5b, instead of a single side. The process conditions used in this experiment are presented in Table 2. The coating stability decreased at a high web speed compared to that at the low web speed, resulting in the formation of a strip-shaped coating layer with irregular uncoated areas instead of the desired one-sided coating layer (Figure 5a).

Therefore, the web speed (production speed) cannot be increased indefinitely when coating occurs at a high speed. To mitigate this problem, the flow rate of the ink ejected from the coater lip can be increased to match the speed of the web. However, this approach increases the CMD speed deviation of the ink ejected from the coater lip, further aggravating the aforementioned CMD thickness variation. Therefore, in this study, we installed the world’s first electrohydrodynamic module in a slot-die coater to electrically influence the ink coated on the web and increase the spreadability of the ink on the web. This approach ensures the filling of gaps in the coated functional layer in the form of strips, owing to excessive web speed.

## 3. Methodology

### 3.1. Meniscus Formation and Spreading Effect

Several variables affect the thickness of the coated functional layer in a slot-die coating system. Typical process parameters that can influence the coated layer thickness include web speed, flow rate, coating gap, and web tension. The coating gap is the vertical height between the coater lip and the web. Based on the assumption that all other conditions are unchanged, the thickness of the coated layer decreases as the web speed increases, and the thickness of the coated layer increases as the flow rate increases; both changes correspond to the continuous equation. When the coating gap increases, the tendency of the thickness to change varies based on the properties of the ink used. For example, in low-viscosity ink, the spreadability is improved by an increase in the gravitational energy, but shear thinning occurs owing to a drop in pressure between the ink and air, which is accompanied by a decrease in coating width [47]. Thus, the shape of the meniscus changes, indicating the tendency of flow between the coater lip and the web. Consequently, the thickness of the coated layer increases slightly. When the last process variable, web tension, is increased, the surface roughness of the web decreases under high tension compared to that under low tension, resulting in a change in surface energy. Therefore, compared to droplets formed on the web under low tension, droplets formed on the web under high tension change the spreadability between the web and ink (lower contact angle), changing the thickness of the coated layer [48].

Changes in the thickness of the coated layer caused by changes in the four process variables (web speed, flow rate, coating gap, and web tension) are caused by changes in the shape of the meniscus formed between the coater lip and the web. First, when the web speed changes, a change occurs in the speed at which the web drags the ink onto the area where the web and ink are in contact at the bottom of the meniscus. Consequently, the shape of the meniscus changes. Second, when the flow rate changes, the volume of the meniscus changes because of a change in the total amount of ink ejected from the coater lip. Third, a change in the coating gap changes the height of the vertical direction from which the ink is ejected. Hence, the potential energy of the ink just before it is ejected is affected, changing the ink spreadability at the moment of contact between the ink and the web, which changes the shape of the meniscus. Finally, if the web tension changes, the surface energy of the web changes, as described above. Consequently, the extent to which the ink spreads on the web at the bottom of the meniscus changes.

**Figure 6:**
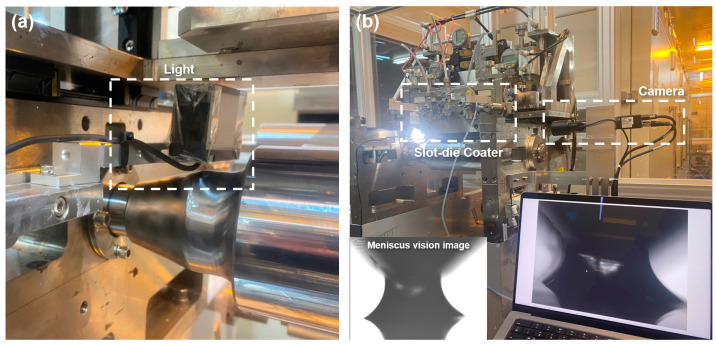
(**a**) Light and (**b**) camera installation for obtaining meniscus vision data.

**Figure 7:**
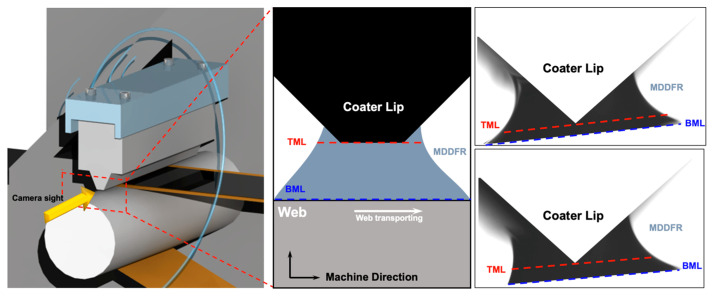
Geometric variables of machine direction meniscus.

**Figure 8:**
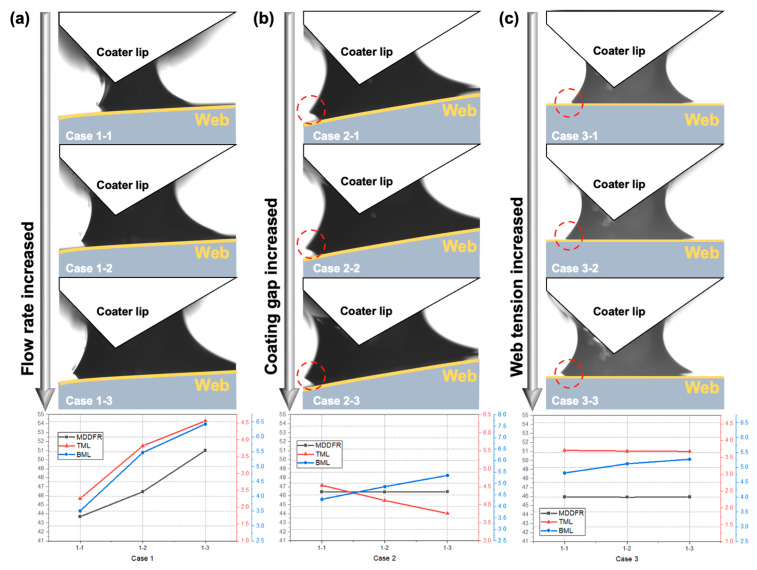
Meniscus geometry variation due to (**a**) flow rate, (**b**) coating gap, and (**c**) web tension.

To verify the meniscus geometry owing to changes in the flow rate and ink spreadability, the changes in meniscus geometry caused by changes in process variables were captured using a camera (STC-MBS163POE, Omrom Sentech Co., Ltd., Yokohama, Japan) that can observe the meniscus. The camera and a light source were installed on opposite sides of the coater, as shown in Figure 6. To determine the extent of the meniscus geometry changes due to changes in flow rate and ink spreadability in the captured meniscus vision images, we defined the machine direction dynamic flow rate (MDDFR), which represents the width of the MD meniscus; top meniscus length (TML), which represents the length of the top and bottom of the meniscus; and bottom meniscus length (BML), as shown in Figure 7. Upon acquiring the meniscus vision data using the camera, the image data were binarized for more accurate comparisons. Subsequently, the MDDFR, TML, and BML for each process condition were derived based on the number of pixels. The inks and webs used in the experiments were the same as those shown in Table 1, and the experimental cases and MDDFR, TML, and BML values according to changes in process parameters are presented in Table 3 and Figure 8a–c.

In Case 1, when the flow rate increases (Figure 8a), the MDDFR, which represents the meniscus volume, increases owing to the increased amount of ink ejected from the coater lip outlet, and the TML and BML also increase simultaneously. As the speed of the web decreases, the shape of the meniscus changes in the same way as in Case 1. In Case 2, the BML increases as the coating gap increases, but the TML decreases similarly due to shear thinning, which results in a nearly constant MDDFR. As the position of the coater lip rises, the gravitational potential energy increases, which in turn increases the spreadability at the web–ink contact (BML). If the web tension is increased, the BML changes because of the change in the web surface properties resulting from the increased tension, which changes the spreadability. In summary, a change in spreadability between the ink and the web causes a change in the meniscus geometry between the coater lip and the web, which in turn changes the coating thickness and thickness variation.

### 3.2. Electrohydrodynamics Module on Slot-Die Coater

To mitigate the impact of the variation in thickness due to deviations in the speed of the ink ejected from the outlet of the coater lip, we enhanced the spreadability between the web and the ink. Previously known factors that primarily affect the spreadability between the web and ink include the surface energy of the web and the surface tension of the ink. However, changing the web or ink requires setting up suitable process conditions for the new web and ink, which is challenging in terms of efficiency.

When the coating gap increases, the vertical distance between the coater lip and the web also increases. Consequently, the gravitational potential energy increases, leading to an increased spreading of the ink upon contact with the web. However, if the coating gap increases, the coating stability degrades, as indicated by Equation (6); therefore, the selection of conditions is limited. Similarly, the use of EHDs increases the ink energy and spreading due to the electrostatic forces. Therefore, the rheological properties of the ink, such as surface tension, could be modulated by controlling the electric field through EHDs. This allowed for the adjustment of the meniscus, thereby maximizing coating stability [49,50]. The introduction of EHDs enabled us to achieve a coating system with higher spreading characteristics compared to traditional coating systems.

**Figure 9:**
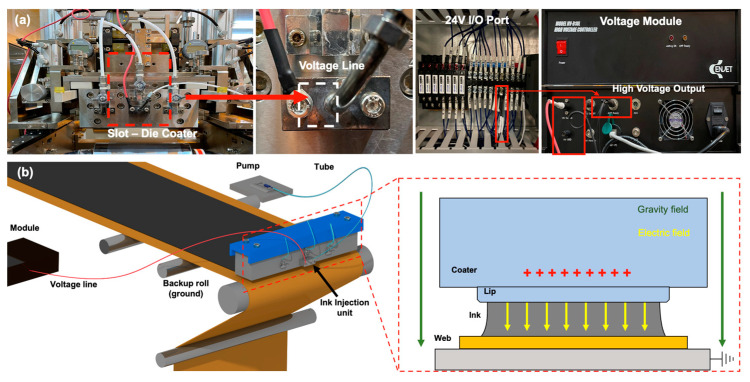
(**a**) EHD module that applies voltage to slot-die coater and (**b**) effect of electrical attraction.

Figure 9a shows the device that applies voltage to the coater. A voltage is applied from the 24 V in/out port to the voltage module, and the amplified voltage from the voltage module is applied through the voltage line to the solution inlet of the slot-die coater. Ground is selected as the coating backup roll, which creates an electrical attraction between the charged ink in the coater and the transferred web on the backup roll, as shown in Figure 9b. Owing to the electrical attraction between the charged ink on the coater lip and the backup roll, the degree of spreading on the web increases during coating. At this point, owing to the speed deviation of the coater outlet, the ink that is heavily coated in the middle of the CMD can be spread to both ends to mitigate the CMD thickness variation.

## 4. Experimental Setup

**Figure 10:**
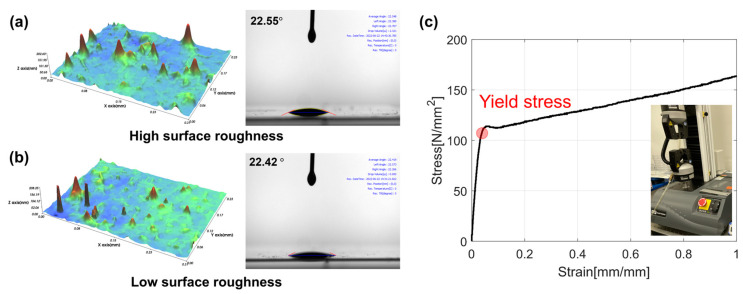
PET film quality related to the coating. (**a**) High surface roughness profile and contact angle, (**b**) Low surface roughness profile and contact angle, (**c**) PET film stress-strain curve.

Experiments were conducted to evaluate the effectiveness of the EHD module in mitigating the CMD thickness variation and its stability in high-speed coating. The web and ink used in the experiment were polyethylene terephthalate film (CD901, Kolon. Inc., Seoul, Republic of Korea) and silver ink (JS-A101A, Novacentrix), respectively. The wettability and printability of the PET film used in this experiment were enhanced via corona surface treatment. Surface roughness is a significant factor affecting coating thickness [10]. To assess the impact of surface roughness variation on coating quality, the surface roughness of PET film was measured at the same locations as in Figure 4c. Figure 10a,b presents the surface roughness profiles for the cases with the highest and lowest surface roughness, along with the results of sessile drop experiments. The surface roughness measurements were recorded as 8.29 nm and 8.05 nm, respectively, with an average of 8.16 nm and a standard deviation of 0.11 nm across all measurement points. The contact angles of the droplets on the two substrates differed by 0.13 degrees, with values of 22.42 and 22.55 degrees, respectively. Since such a difference leads to changes in the coated layer of only a few nanometers, this study can disregard the influence of surface roughness variation on coating thickness [20]. Additionally, as shown in Figure 10c, the elastic modulus of the material was 3.19 GPa, and the yield strength was 109.7 MPa, as confirmed by the stress–strain curve. The tension applied to the PET film in the R2R system ranged from 10% to 25% of the material’s yield strength [51,52]. Considering the yield strength of the PET used in the experiment, a tension of 2 kgf was applied in this study. Furthermore, to observe sensitive changes induced by EHD effects, a conductive ink, sensitive to electrical influences, was utilized. The properties of the ink and solvent used are summarized in Table 1. Due to the high conductivity of the ink employed, the dielectric polarization of the solvent has a lesser impact on the electrical attraction than silver ink [53].

The experimental details presented in Table 4 were selected to verify the effectiveness of mitigating the CMD thickness variation. The drying temperature was set according to the ink characteristics, and the operating speed was set according to the minimum drying time and the length of the dryer. The magnitude of the voltage was selected in the range of voltages that do not cause sparking at a coating gap of 100 μm by considering the voltage threshold. The voltage applied to the slot-die coater was set to 0 V, 1 kV, and 2 kV, whereas all other process variables were fixed. The coating meniscus geometry based on the magnitude of the applied voltage was photographed to confirm the MDDFR and BML. The thickness of the CMD was measured using a scanning electron microscope (SEM, SU8010, Hitachi Inc., Tokyo, Japan). The measurement positions of the samples were the same as those shown in Figure 4c.

The experiments detailed in Table 5 were conducted to verify the coating stability during high-speed production on the EHD slot-die coater. With no voltage applied, the web speed was increased (from 1.0 to 3.0 mpm) to form an irregular strip rather than a coating layer. In this state, the voltage was applied to the slot-die coater to confirm that the strip was filled with CMD due to the high ink spreadability. The experiment was performed with both conductive and non-conductive inks for universality.

## 5. Results and Discussion

### 5.1. Effect of EHDs on Coating Meniscus

**Figure 11:**
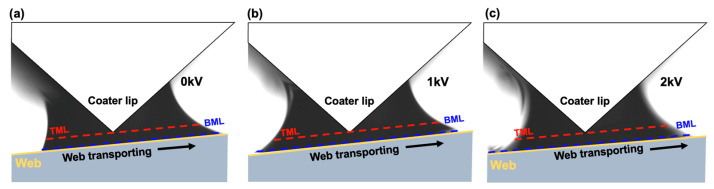
Meniscus geometry at applied voltages of (**a**) 0 kV, (**b**) 1 kV, and (**c**) 2 kV.

The MDDFR, TML, and BML were derived for each condition by imaging the meniscus while performing slot-die coating of silver ink under the conditions listed in Table 4. The coating was performed for 10 min in total (coated length = 5 m), and 100 images were acquired per minute. The averages and deviations of MDDFR, TML, and BML are presented in Table 6. The change in the meniscus geometry over time was hardly observed because the coating meniscus was stabilized by capturing images 1–2 min after the ink was ejected from the coater lip [54,55]. Furthermore, the deviations in MDDFR, TML, and BML over time were minimal because the tension disturbances that might occur in the coating section of the R2R system, such as eccentricity and syringe pulsation, were minimized. Figure 11a–c shows the meniscus geometry when the applied voltage was 0 kV, 1 kV, and 2 kV, respectively. Table 6 presents the calculation results for MDDFR, TML, and BML under each condition.

**Figure 12:**
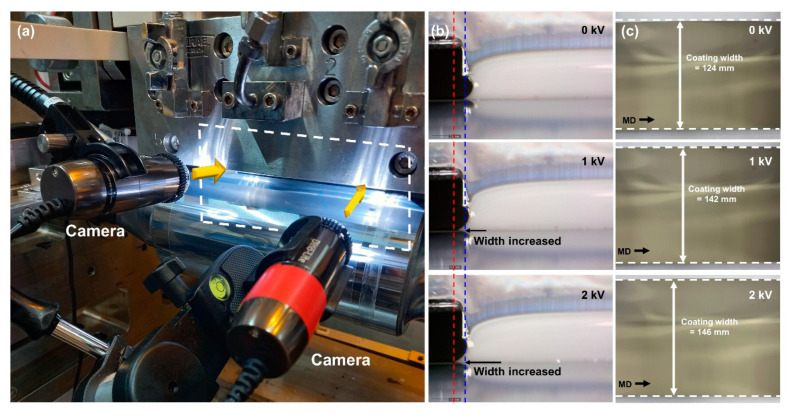
(**a**) Camera installation for CMD meniscus, (**b**) spreading effect at CMD, and (**c**) width of coated layer.

**Figure 13:**
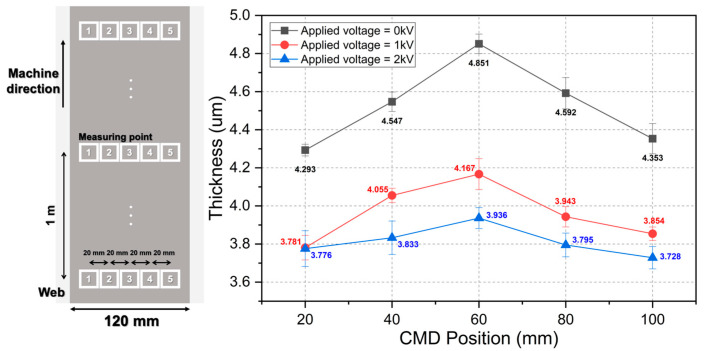
Thickness variation according to applied voltage.

These results show that the MDDFR was approximately constant in both the applied and unapplied cases. Furthermore, the TML decreased while the BML increased. The increase in TML indicates that the spreadability between the ink ejected from the coater lip and the web increased because of electrical attraction. Here, the larger increase in BML compared to the decrease in TML with constant MDDFR indicates that the coating meniscus was expanded by the CMD. Figure 12a shows the camera (AM7915MZLT, Dino-Lite. Inc., New Taipei City, Taiwan) installed to confirm the expansion of the CMD meniscus. Figure 12b shows the CMD meniscus photographed with each voltage applied, which shows that the spreadability of the CMD meniscus increases with the voltage, resulting in an increase in width (blue line to red line). Figure 12c shows the result of measuring the width of the formed silver-coated layer, which shows that the width of the coated layer increased by 12.7% when the applied voltage was 1 kV. Therefore, the increase in ink spreadability owing to electrical attraction occurs simultaneously in the CMD and MD. To analyze the effect of this increase in spreadability on the CMD thickness profile of the coated layer, the thickness of the coated layer for each condition is shown in Figure 13. The average thicknesses of the coated layer were 4.53 μm, 3.96 μm, and 3.81 μm at applied voltages of 0 kV, 1 kV, and 2 kV, respectively. As the spreadability between the ink and the web improved because of electrical attraction, the thickness variation of the CMD decreased by the proportion of ink spreading to the CMD (124 mm to 142 mm to 146 mm). The thickness variation of the CMD was 0.197 μm, 0.138 μm, and 0.070 μm, respectively. By increasing the voltage from 0 kV to 1 kV, the thickness variation was alleviated by approximately 29.9% by spreading the coating liquid that was heavily deposited at the center of the web to both ends through the speed deviation of the coater lip outlet. The thickness variation was reduced by up to 64.5% by further increasing the voltage.

### 5.2. Coating Bead Stabilization

**Figure 14:**
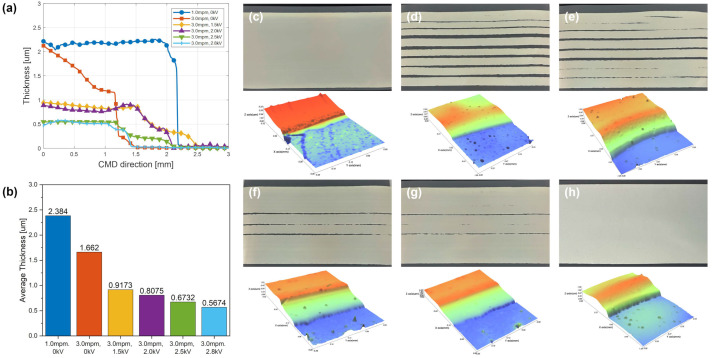
(**a**) Thickness profile and (**b**) average thickness variation according to applied voltage and web speed: (**c**) 1.0 mpm, 0 kV, (**d**) 3.0 mpm, 0 kV, (**e**) 3.0 mpm, 1.5 kV, (**f**) 3.0 mpm, 2.0 kV, (**g**) 3.0 mpm, 2.5 kV, and (**h**) 3.0 mpm, 2.8 kV.

The experimental results shown in Figure 14 confirm the stability of high-speed coating with the EHD module attached. Figure 14a,b shows the thickness profile and average thickness for each case in Table 5. Figure 14c shows a stable coated layer at a web speed of 1.0 mpm. Figure 14d shows that when the speed increased to 3.0 mpm, an irregular strip-shaped coated layer was formed because the flow rate was insufficient to cover the speed of the conveyed web. Figure 14e–h shows the cases where the voltage was increased under the same conditions as in Figure 14d. As the voltage increased, the spreadability of the CMD increased, causing the voids in the irregular strips to be filled gradually until the strips were all filled at a certain voltage. In addition, the average thickness of the coated layer decreased as the voids were filled. If the operating speed increases under the same conditions (Figure 14c,d), the flow rate at the outlet of the coater is not sufficient compared to the web speed, so the thickness decreases significantly. At this point, increasing the voltage (Figure 14e–h) results in a slight decrease in thickness as the voids fill, owing to the spreadability change at the same flow rate. Consequently, a stable coated layer was formed at high speeds by combining the EHD module with the defect of a partly formed coated layer when a coating is normally performed at a speed of 3.0 mpm. Thus, the production speed can be increased sufficiently if the appropriate voltage is set and applied (300% in this case).

### 5.3. Electrical Conductivity of Ink

**Figure 15:**
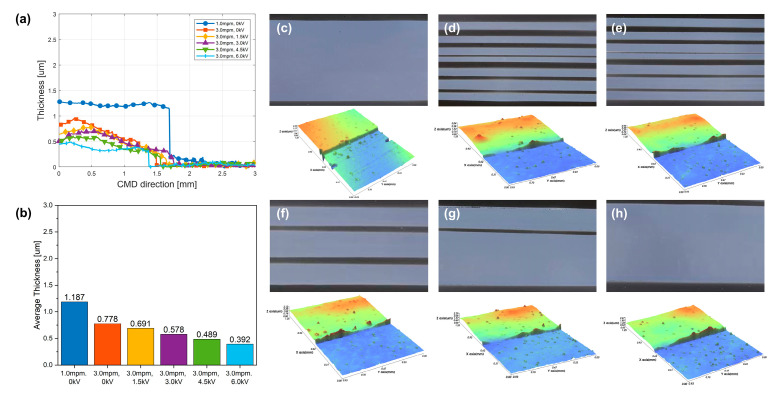
Strip variation at dielectric ink coating (**a**) Thickness profile and (**b**) average thickness variation according to applied voltage and web speed: (**c**) 1.0 mpm, 0 kV, (**d**) 3.0 mpm, 0 kV, (**e**) 3.0 mpm, 1.5 kV, (**f**) 3.0 mpm, 3.0 kV, (**g**) 3.0 mpm, 4.5 kV, and (**h**) 3.0 mpm, 6.0 kV.

**Figure 16:**
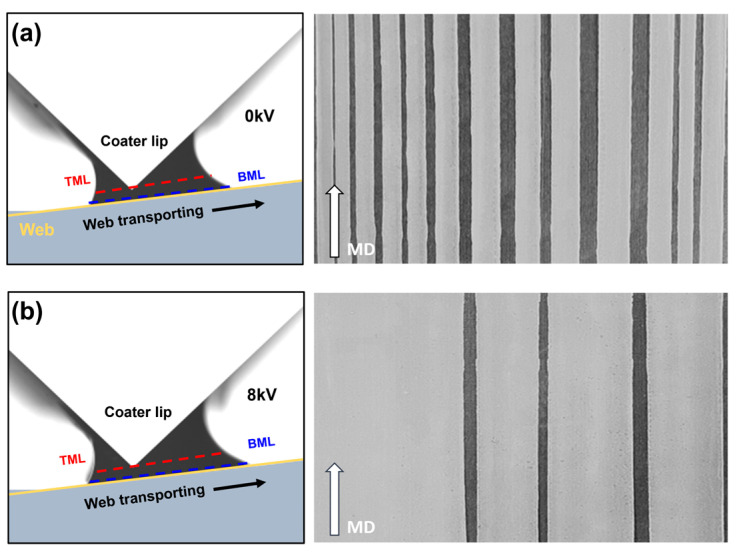
EHD effects in solutions with low dielectric constant (**a**) case without EHDs: 3.0 mpm, 0 kV, (**b**) case with EHDs: 3.0 mpm, 8 kV.

The experiments performed as described in Section 5.2 using conductive silver ink were repeated using non-conductive dielectric ink (PD-100, Paru.Inc., Suncheon, Republic of Korea). The properties of the inks are listed in Table 7. In this case, compared with the dielectric constant of the dielectric ink, which is approximately 20, the diethylene glycol monoethyl ether acetate used as a solvent has a dielectric constant of approximately 2.54. Therefore, the electrical influence of the solvent’s polarity compared with that of the ink is minor, allowing it to be disregarded. Figure 15a,b shows the thickness profile and average thickness in each case. Figure 15c–h shows that the width of the coated layer increased with the conductive ink when the applied voltages were 0, 1.5, 3.0, 4.5, and 6 kV, respectively. This phenomenon is due to dielectric polarization. When a high voltage is applied to the dielectric liquid filled in the coater reservoir, the ink inside the coater is charged by the polarization phenomenon and ejected to the lip of the coater to form a meniscus. Moreover, the phenomenon increases spreadability. However, the extent of spreading is significantly reduced in non-conductive ink compared to the case of conductive ink. To achieve the same increase in spreadability, the magnitude of the applied voltage must also be sufficiently large to cause dielectric polarization. Furthermore, the dielectric constant of the ink also influences EHD effects. When the dielectric constant is low, dielectric polarization occurs less readily, leading to a reduction in the EHD effects. To confirm this, an EHD application experiment was conducted using a diethylene glycol monoethyl ether acetate solution with a dielectric constant of 2.54, employing the same process speed of 3 mpm. Figure 16a shows the case without applying EHDs, and Figure 16b depicts the case with an applied voltage of 8 kV, which is the maximum voltage that the EHD module can apply. Upon applying EHDs, variations in the boundary meniscus layer (BML) and the filling of voids in the coating layer to a certain extent were observed. Thus, the presence of EHD effects was confirmed even with a low dielectric constant. However, strip coating was still observed, and the stabilization of a complete coating meniscus was not achieved, indicating that a higher voltage input is required.

## 6. Conclusions

This study mitigated the coating layer thickness variation caused by speed deviation in the coater lip outlet of an R2R system, which is an inherent limitation of the R2R slot-die coating system. Electrical attraction was applied by combining an EHD module with the coater to address the variation in thickness. When a high voltage was applied to the coater to charge the ink or induce electric polarization, the ink ejected from the coater lip received an electric force potential energy, in addition to the gravitational potential energy. Thus, the spreadability of the meniscus formed when the ink and web meet increases. This spreadability increases in both the MD and CMD, which was demonstrated by observing the meniscus geometry and volume experimentally. When the spreadability of both the CMD and MD increases, the thickly coated ink at the center of the web spreads to both ends. This mitigates the variation in the CMD thickness and increases the width, resulting in the formation of an ultra-thin, uniform, coated layer that is smaller and more uniform than the conventional thickness. The thickness variation of CMDs was reduced by up to 64.5% and was mitigated by combining EHD modules based on a conductive ink. The irregular strip formation owing to meniscus instability that occurs during high-speed coating could be suppressed using the spreadability of the CMD. Therefore, the speed at which stable coating can be achieved was increased by approximately 300%, which can further improve the high-speed productivity of an R2R system. Even when using non-conductive ink, the spreading effect due to the EHD module was observed, albeit to a lesser extent compared to conductive ink. Additionally, it has been confirmed that the spreading effect is influenced by the dielectric constant of the ink. However, there was a limitation in determining the precise extent of the increase in spreadability with respect to the applied voltage. In future work, the amount of thickness reduction and width increase when a voltage is applied will be modeled mathematically. We anticipate that the width and thickness of the ultra-thin, uniform, coated layer to be fabricated can be determined by predicting the thickness decrease and increase in width based on the applied voltage. Additionally, experiments will be conducted to expand the research by applying higher voltages to ensure the coating stability of inks with low dielectric constants.

## Figures and Tables

**Table 1:** Process conditions and ink and substrate properties.

	Parameter	Unit	Value
Process conditions	Shim plate thickness	μm	100
	Coating width	mm	120
	Web speed	m/min	0.5
	Coating gap	μm	100
	Flow rate	mL/min	0.5
Ink properties (JS-A101A, NOVACENTRIX.Inc., Austin, TX, USA)	Ink viscosity	cP	10
	Weight percent	%	40
	Conductivity	MS/m	12.82
	Surface tension	mN/m	19
Solvent properties (DI Water)	Ink viscosity	cP	1
	Assay	%	99
	Surface tension	mN/m	73
	Relative dielectric constant	−	80
Substrate properties (CD901, KOLON.Inc., Seoul, Republic of Korea)	Thickness	μm	100
	Width	mm	150
	Young’s Modulus	GPa	3.19
	Yield stress	MPa	109.7

**Table 2:** Process conditions for stable and unstable coatings.

Parameter	Unit	Value
Shim plate thickness	μm	100
Coating width	mm	120
Web speed	m/min	5 (Stable coating) 10 (Unstable coating)
Coating gap	μm	100

**Table 3:** Experimental cases and results of analyzing the effect of flow rate and spreadability on meniscus geometry.

Case	Flow Rate [mL/min]	Coating Gap [μm]	Web Tension [kgf]	MDDFR	TML	BML
1–1	3	200	3.7	43.69	2.26	3.51
1–2	4			46.44	3.82	5.47
1–3	5			51.02	4.56	6.43
2–1	4	50	2.7	46.44	4.64	4.31
2–2		100		46.41	4.12	4.86
2–3		150		46.45	3.76	5.35
3–1	4	200	0.7	45.96	3.71	4.81
3–2			2.7	45.92	3.69	5.12
3–3			4.7	45.95	3.68	5.27

**Table 4:** Experimental cases for analyzing the EHD effect on CMD thickness variation.

Process Variable	Unit	Value
Coating gap	μm	100
Web speed	m/min	0.5
Ink viscosity	cP	10
Flow rate	mL/min	1.5
Drying temperature	℃	90
Operating tension	kgf	2
Voltage	kV	0, 1, 2

**Table 5:** Experimental cases for analyzing the EHD effect on coating stability at high speeds.

Process Variable	Unit	Case 1	Case 2	Case 3	Case 4	Case 5	Case 6
Ink viscosity	cP	10	10	10	10	10	10
Coating gap	μm	100	100	100	100	100	100
Flow rate	mL/min	1.5	1.5	1.5	1.5	1.5	1.5
Drying temperature	℃	90	90	90	90	90	90
Operating tension	kgf	2	2	2	2	2	2
Web speed	m/min	1.0	3.0	3.0	3.0	3.0	3.0
Voltage	kV	0	0	1.5	2.0	2.5	2.8

**Table 6:** Effect of applied voltage on meniscus geometry.

Applied Voltage	0 kV	1 kV	2 kV
MDDFR (std)	38.74 (0.02)	38.79 (0.03)	38.76 (0.02)
TML (std)	1.35 (0.01)	1.30 (0.01)	1.28 (0.03)
BML (std)	1.88 (0.01)	2.39 (0.01)	2.85 (0.01)

**Table 7:** Properties of dielectric ink.

	Parameter	Unit	Value
Ink properties (PD-100, Paru.Inc.)	Ink viscosity	cP	30
	Weight percent	%	40
	Surface tension	mN/m	32
	Relative dielectric constant	−	20
Solvent properties (diethylene glycol monoethyl ether acetate, Duksan.Inc., Chungbuk, Republic of Korea)	Ink viscosity	cP	2.8
	Assay	%	99
	Surface tension	mN/m	31.7
	Relative dielectric constant	−	2.54

## Data Availability

Data are contained within the article.
